# Value of Ultrahigh‐Resolution Photon‐Counting Detector Computed Tomography in Cardiac Imaging

**DOI:** 10.1111/echo.70100

**Published:** 2025-02-13

**Authors:** Dmitrij Kravchenko, Muhammad Taha Hagar, Milan Vecsey‐Nagy, Giuseppe Tremamunno, Bálint Szilveszter, Borbála Vattay, Emese Zsarnóczay, Sámuel Beke, Pál Maurovich‐Horvat, Tilman Emrich, Akos Varga‐Szemes

**Affiliations:** ^1^ Department of Radiology and Radiological Science Medical University of South Carolina Charleston USA; ^2^ Department of Diagnostic and Interventional Radiology University Hospital Bonn Bonn Germany; ^3^ Quantitative Imaging Lab Bonn (QILaB) University Hospital Bonn Bonn Germany; ^4^ Department of Diagnostic and Interventional Radiology Medical Centre Faculty of Medicine University of Freiburg University of Freiburg Freiburg im Breisgau Germany; ^5^ Heart and Vascular Center Semmelweis University Budapest Hungary; ^6^ Department of Medical Surgical Sciences and Translational Medicine Sapienza University of Rome – Radiology Unit – Sant'Andrea University Hospital Rome Italy; ^7^ Medical Imaging Center Semmelweis University Budapest Hungary; ^8^ Department of Diagnostic and Interventional Radiology University Medical Center of the Johannes Gutenberg‐University Mainz Germany; ^9^ German Centre for Cardiovascular Research Partner Site Rhine‐Main Mainz Germany

**Keywords:** cardiac CT, coronary CT, photon‐counting detector CT, ultrahigh‐resolution

## Abstract

It was only fitting that when computed tomography (CT) was celebrating its 50th birthday since its maiden scan in 1971, it was also entering into a new generation in 2021 with the Food and Drug Administration's approval of the first photon‐counting detector (PCD)‐CT. As non‐invasive cardiac imaging is evolving into an ever more important medical field, the introduction of this new technology promises a slew of improvements over energy‐integrating detector (EID)‐CTs, most importantly improved spatial resolution in the form of ultrahigh‐resolution (UHR) imaging, reduced radiation exposure, and routinely acquired spectral information. Spatial resolution has historically been a key hurdle for cardiac CT, especially for coronary imaging where structures in the realm of 2 mm need to be assessed. Initial reports on the use of PCD‐CT in cardiac imaging so far have been promising, but many questions ranging from standardized scan protocols to evidence‐based recommendations remain. The aim of this review is to discuss the currently available literature regarding the use of UHR PCD‐CT for cardiac imaging and explore if it has led to changes in guidelines or patient workflows.

## Graphical abstract

Photon‐counting detector computed tomography (PCD‐CT) represents a big leap forward in non‐invasive cardiac assessment over conventional standard resolution energy‐integrating detector (EID)‐CTs. This new detector technology enables ultrahigh‐resolution (UHR) imaging at resolutions of up to 0.2 mm. Additional filtering of electronic noise and photon energy discrimination allow PCD‐CT to image patients with a high calcium burden, assess in‐stent patency, improve stenosis quantification and accuracy, as well as the visualization of small diameter vessels.

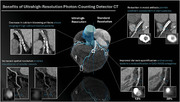

**Abbreviations**
CAC: coronary artery calcium
CAD: coronary artery disease
CAD‐RADS: coronary artery disease reporting and data system
CCS: chronic coronary syndrome
CCTA: coronary computed tomography angiography
CNR: contrast‐to‐noise ratio
CT: computed tomography
CT‐FFR: computed tomography fractional flow reserve
EAT: epicardial adipose tissue
ECG: electrocardiogram
EID: energy‐integrating detector
FAI: fat attenuation index
ICA: invasive coronary angiography
ICC: intraclass correlation coefficient
IQR: interquartile range
MACE: major adverse cardiovascular events
PCAT: pericoronary adipose tissue
PCD: photon‐counting detector
QCA: quantitative coronary angiography
SNR: signal‐to‐noise ratio
TAVR: transcatheter aortic valve replacement
UHR: ultrahigh‐resolution
VMI: virtual monoenergetic images
VNC: virtual non‐contrast

## 1 Introduction

Computed tomography (CT) has emerged as a staple in medical infrastructure and diagnostic imaging since its maiden scan in the 1970s [1]. Its absence would be unimaginable in current hospital workflows and many specialties, ranging from neurology to cardiology, rely heavily on it to ensure adequate and modern patient care. As heart disease remains the most common cause of death worldwide [2], it has become pertinent to develop accurate and reliable tools for its diagnosis. Coronary CT angiography (CCTA) has evolved into one of the corner stones of diagnostic workups for the evaluation of cardiovascular diseases such as coronary artery disease (CAD) and congenital heart disease. Throughout its wide adoption, CCTA has been incorporated into several international guidelines as the diagnostic test of choice in patients with chronic chest pain, and intermediate pretest likelihood [3, 4]. In 2022, the large international, multicenter, randomized DISCHARGE trial demonstrated no differences in outcome for patients who underwent either CCTA or invasive coronary angiography (ICA) for diagnostic work‐up at a follow‐up of 3.5 years [5]. At this point CCTA was not only able to rule out obstructive CAD with a similar clinical outcome as ICA but could also provide much more additional data such as plaque composition, evaluation of epicardial and pericoronary fatty tissues, and functional data.

Unfortunately, conventional energy‐integrating detector (EID)‐CTs suffer from drawbacks such as limited spatial resolution to resolve small structures, deteriorated image quality due to electronic noise, and blooming artifacts caused from high attenuation material, keeping them from surpassing ICA as the modality of choice for high‐risk cases. Recently, the development and clinical approval of a new type of CT technology termed photon‐counting detector (PCD)‐CT has brought a myriad of changes to the world of CT including suppression of electronic noise, ultrahigh‐resolution (UHR) defined as imaging on detector elements with a size of under 0.25 mm [6], a potential decrease in radiation dose and artifacts, multi‐energy imaging with always‐on spectral information, and improved contrast‐to‐noise ratios (CNR) [7]. This review will discuss the current state of cardiac PCD‐CT with a focus on UHR applications, its advantages and limitations, as well as its outlook.

## 2 Technical Aspects

The key difference between EID‐ and PCD‐CTs lies in their respective detector technologies. In conventional EID‐CTs, x‐rays are first converted into visible light photons via scintillators which are then detected by a photodiode and finally converted to an electronic signal [8]. The use of small highly reflective septa is necessary to separate the individual EID elements to avoid cross‐talk. Unfortunately, they also limit the size of individual detector elements and in turn the highest achievable spatial resolution. Furthermore, septa additionally increase radiation exposure due to suboptimal geometric dose efficiency. The signal strength of EIDs consists of the integral of all current pulses detected during a specific measurement time, so that individual photon energies are not recorded. As a result, x‐ray photons with higher energy contribute disproportionately to the signal. This leads to a lower CNR as low‐energy x‐ray photons carry the majority of contrast information [9, 10].

**FIGURE 1:**
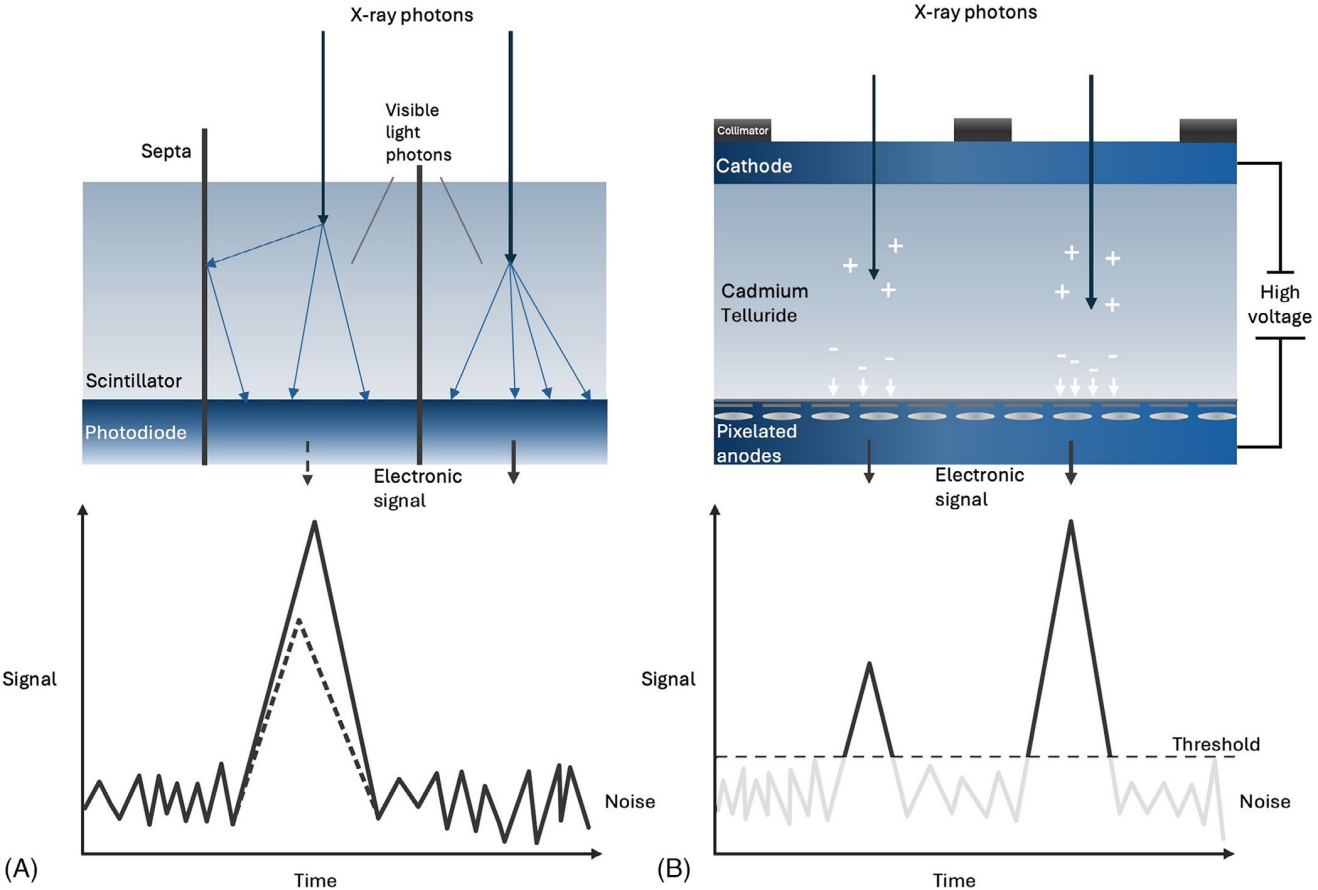
Comparison of energy‐integrating detector CT (EID)‐CT (A) and photon‐counting detector (PCD)‐CT (B) technologies. In EID‐CTs x‐ray photons must first be converted into visible light before they can be detected using a photodiode which cannot discriminate between low‐ and high‐energy photons. In PCD‐CTs each x‐ray photon gets quantified separately after being absorbed through a semiconductor material.

PCDs are fundamentally built different from the ground up. Through the use of a semiconductor, such as, for example, cadmium telluride or silicone, and a high current, PCD‐CTs generate a cloud of negative and positive electrons allowing for the direct conversion of x‐ray photons to electronic signals without the intermediary step of first having to convert them into visible light. This direct signal conversion combined with the lack of necessity for the integration of septa allows smaller detector sizes. Moreover, detector sub‐pixel units can be read‐out separately, which ultimately increases spatial resolution allowing for UHR imaging [8, 9]. A schematic representation comparing EID‐CT and PCD‐CT is depicted in Figure 1.

These critical differences in detector technology led to three main advantages for PCD‐CTs. Firstly, the smaller detector units lead to increased spatial resolution and capabilities for UHR imaging with a slice thickness as thin as 0.2 mm, significantly improving on the 0.4–0.5 mm slice thickness available on most EID‐CTs [11]. Secondly, the ability to register individual x‐ray photon energies allows the binning of energy levels which enables filtering of electronic noise. And lastly, energy‐binning allows spectral image acquisitions, such as virtual monoenergetic imaging (VMI).

### 2.1 Limitations of PCD‐CT Technology

**FIGURE 2:**
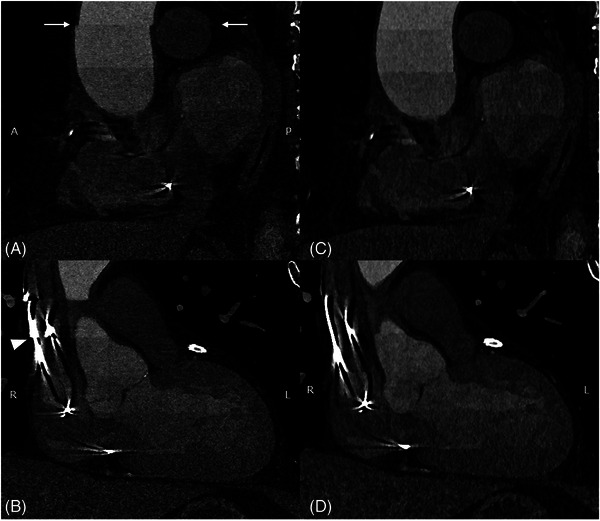
Sagittal (A) and coronal (B) multiplanar reconstructions of an 80‐year‐old male using UHR PCD‐CT (0.2 mm slice thickness, quantum iterative reconstruction strength level of 4, window level 300, width 900). On the sagittal view, a stairstep artifact can be seen affecting the ascending aorta and the right pulmonary artery (arrows). On the coronal views, the artifact can be readily observed affecting the partly imaged pacemaker leads (arrowhead). After the application of a special cardiac stack correction software, proper alignment has been restored on both sagittal (C) and coronal (D) views.

**FIGURE 3:**
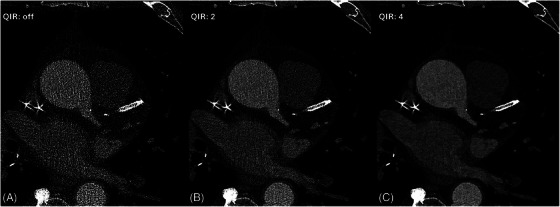
Axial coronary computed tomography angiography of an 80‐year‐old male using UHR PCD‐CT (0.2 mm slice thickness, sharp Bv64 kernel, window level 300, width 900) using different strengths of quantum iterative reconstruction (QIR). With no use of QIR images appear very noisy; stent assessment is not possible (A). With increasing strength of QIR (B,C) image impression becomes less noisy allowing for better assessment of smaller structures.

As with any budding new technology, there are still some hurdles to overcome. For one, there is currently only one Food and Drug Administration‐approved PCD‐CT scanner available on the market (NAEOTOM Alpha, Siemens, Forchheim, Germany), although other vendors have prototypes in development [12, 13]. A brief summary of current PCD‐CT challenges is listed below. For a comprehensive review, the work by Flohr et al. is recommended [9]. Figure 2 provides an example of a misalignment artifact, commonly observed on sequential PCD‐CT acquisitions and a possible solution to it in the form of cardiac stacked correction. Another limitation of UHR PCD‐CT is the noise penalty imparted by thinner slice selections and sharper kernels. Figure 3 demonstrates how iterative reconstructions can help mitigate the higher noise observed on PCD‐CT imaging.

#### 2.1.1 Pulse Pileup

Successive x‐rays accumulate to a higher energy when incidents occur within a short timeframe. This results in increased image noise, as fewer photons contribute effectively to image quality. Additionally, this also leads to a degradation of energy resolution, as the recorded energies no longer correspond to individual photons.

#### 2.1.2 Cloud Sharing

When x‐rays are absorbed near pixel borders, the resulting current pulses are divided between neighboring detector cells. This causes a high‐energy x‐ray photon to be incorrectly counted as multiple lower‐energy hits leading to detection on both cells but only with partial energy. The result is a decrease in spatial resolution and CNR.

#### 2.1.3 K‐escape

When incident x‐rays at energies close to the K‐edges of the detector material eject K‐electrons from the detector material, the vacant K‐shells are promptly refilled, releasing characteristic x‐rays at the K‐shell fluorescence energy. These can be then reabsorbed and counted either in the same detector cell or in adjacent cells, a process known as K‐escape. Consequently, the original x‐rays at the primary interaction site lose energy and are recorded at a lower energy.

## 3 Current Applications of UHR PCD‐CT

### 3.1 Coronary Artery Calcium Scoring

The coronary artery calcium (CAC) score was developed in the early 90s and has since evolved into a reliable, reproducible, quantitative method to assess cardiovascular risk [14, 15]. It has been shown to be a good predictor of the likelihood of major adverse cardiovascular events (MACE) and a better predictor of coronary heart disease compared to conventional biomarkers like age, sex, and body mass index [16, 17], recently making its way into European guidelines [18]. The standardization of CAC scoring protocols was followed in 2007 with the agreement of scan and reconstruction parameters (e.g., tube potential of 120 kVp and a slice thickness of 3 mm) [19, 20]. PCD‐CT could help to more accurately quantify CAC burden via increased spatial resolution, decreased calcium blooming, and material decomposition.

Initial phantom [21] and cadaveric trials [22] showed that existing protocols for CAC scoring could be applied to new PCD‐CT systems yielding similar results when a thinner slice thickness or sharper kernels were applied. In vivo studies proceeded to demonstrate the benefits of PCD technology by showing that PCD‐CT CAC scoring resulted in higher‐quality images and reduced radiation doses without compromising diagnostic image quality [23, 24]. A combined phantom and intra‐individual comparison confirmed that PCD‐CTs can be used for CAC scoring and may even provide more accurate CAC quantification based on concurrent phantom findings [25]. CAC volume measurements on a first‐generation dual source PCD‐CT were significantly lower (66.1 ± 1.6%) compared to an EID‐CT (77.2 ± 0.5%), with the PCD‐CT measurements being more in line with the phantom. The routine acquisition of spectral data allowing for the reconstruction of VMIs and calcium‐preserving virtual non‐contrast images (VNC) on PCD‐CT systems may replace the need for non‐contrast enhanced acquisitions on cardiac CT in the future through the use of VNCs [26], but that topic is outside of the scope of this review.

### 3.2 Coronary CT Angiography

**FIGURE 4:**
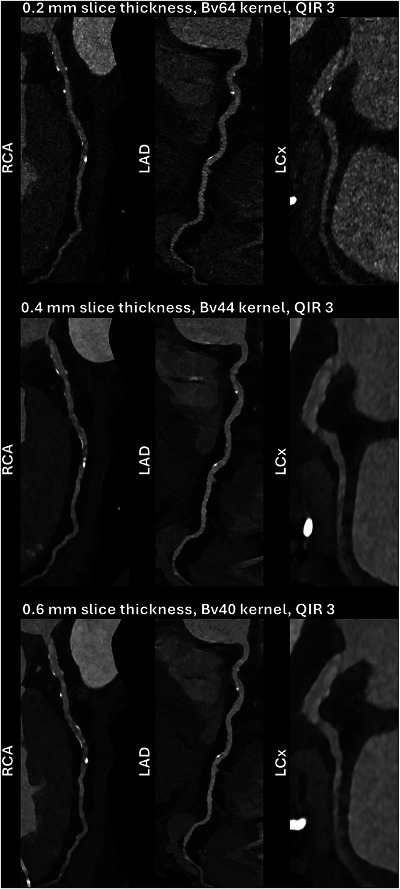
Comparison of the effects of slice thickness and convolution kernel on image impression using photon‐counting detector coronary computed tomography angiography in a 64‐year‐old male with a coronary artery calcium score of 252 Agatston units. LAD, left anterior descending artery; LCx, Left circumflex artery; QIR, quantum iterative reconstruction; RCA, right coronary artery.

Non‐invasive CCTA has been increasingly looking to replace diagnostic ICA as the reference standard in stable patients with angina pectoris. Although ICA allows unparalleled temporal resolution and assessment of hemodynamics, it does not provide information about plaque morphology or composition without the use of intravasal imaging such as optical coherence tomography or intravascular ultrasound. Especially in non‐ or partly‐calcified lesions, high‐risk plaque features such as spotty calcifications, presence of a fibrous cap, high lipid content, and positive remodeling have been identified as hallmarks of vulnerable plaques [27, 28]. The DISCHARGE trial found that the pretest probability of CCTA and ICA for risk of MACE was comparable, while the frequency of major procedure‐related complications was much lower for CCTA than ICA [5]. Current EID‐CTs are hampered by calcium blooming which leads to an overestimation of vessel stenosis [29, 30] and beam hardening artifacts that limit diagnostic assessment of coronary stents [31]. Furthermore, image quality is affected by numerous acquisition and reconstruction settings such as tube potential, convolution kernel and kernel sharpness, and slice thickness. Sharper kernels and thinner slices contribute to a better spatial resolution but at the cost of increased noise and a reduced CNR. An example comparing the effects of slice thickness and kernel sharpness on image impression in the same patient is depicted in Figure 4. The dual‐source configuration of CT scanners also plays an important role in image quality. A study of 30 patients who underwent UHR coronary PCD‐CCTA had their data reconstructed using information from only a single source (temporal resolution: 125 ms) and the usual dual‐source data (66 ms) [32]. It found that image quality was significantly better with a higher temporal resolution due to less motion artifacts, better vessel delineation and sharpness, and less blooming.

#### 3.2.1 Stenosis Quantification and Accuracy

It is known that CCTA has limited value in patients with a high burden of coronary calcium (CAC scores >1000 Agatston units) due to severe blooming artifacts obscuring the coronary lumen [33]. The introduction of PCD‐CTs into clinical routine with its higher spatial resolution and spectral imaging promised to address these EID‐CTs shortcomings [30, 34].

An initial phantom study comparing UHR and standard resolution demonstrated the superiority of UHR scanning parameters for stenosis accuracy measurements [35]. In this study, UHR consistently measured more accurate 25/50% stenoses at different heart rates than standard resolution (e.g., 50% stenosis, 51.0% vs. 60.3% at 60 bpm). Later human investigations went on to observe excellent diagnostic accuracy of PCD‐CT for identifying obstructive CAD when compared to the reference standard of ICA, with a sensitivity of 100% (95% CI [96.07%–100.00%]) and specificity of 87.50% (95% CI [81.53%–92.09%]) [36]. One study, consisting of a phantom and in vivo measurements with ICA as a reference, found that decreased blooming artifacts led to decreased percentage diameter stenoses on high‐resolution PCD‐CT for calcified and mixed plaques [37]. In another study by Halfmann et al., the authors observed a decrease in median percentage diameter stenosis with an increase in spatial resolution when using UHR PCD‐CT for calcified lesions [38]. Similar to the previously mentioned publications, calcified plaques benefited the most from UHR (median percentage stenosis 26.7% [IQR, 18.6%–44.3%] compared to standard resolution 41.5% [IQR, 27.3%–58.2%] or high resolution 34.8% [IQR, 23.7%–55.1%]; *p* < 0.001), whereas partially calcified and non‐calcified plaques did not seem to share the same benefits (*p* ≥0.88). In turn, the use of UHR led to a lower reclassification of CAD‐RADS score.

Earlier this year, Koons et al. enrolled 23 patients with a total of 34 stenoses to undergo EID‐ and UHR PCD‐CT on the same day and performed diameter percentage stenosis measurements [39]. Their findings showed that stenoses decreased on average by 11% going from EID‐ to PCD‐CT, resulting in a downgrading of stenosis severity in 13 out of 34 stenoses (38%).

Vecsey‐Nagy et al. conducted an intraindividual comparison of percentage diameter stenoses between EID‐ and PCD‐CT in 49 patients with 278 plaques. PCD‐CT had a higher correlation with quantitative coronary angiography (QCA) (0.91 [95% CI, 0.85–0.95]) compared to EID‐CT and QCA (0.83 [95% CI, 0.71–0.90]), leading to smaller stenosis measurements for calcified plaques (PCD‐CT vs. EID‐CT: 45.1 ± 20.7 vs. 54.6 ± 19.2%; *p* < 0.001) and partially calcified lesions (44.3 ± 19.6 vs. 54.9 ± 20.0%; *p* < 0.001), but not for non‐calcified lesions (39.1 ± 15.2 vs. 39.0 ± 16.0%; *p* = 0.98). The higher agreement of PCD‐CT with QCA led to a reclassification of the Coronary Artery Disease Reporting and Data System (CAD‐RADS) score in 49% patients to a lower score [40].

Hagar et al. conducted a prospective study in patients with severe aortic valve stenosis indicated for transcatheter aortic valve replacement (TAVR) who underwent clinically indicated PCD‐CCTA and ICA validation [41]. UHR PCD‐CT had an excellent per‐patient sensitivity (96%, 95% CI 79–100), specificity (84%, 95% CI 58–95), and accuracy (88%, 95% CI 78–95) for the detection of obstructive CAD (stenosis > 50%). More importantly, accuracy for patients with severe coronary calcifications (Agatston score >1000) was 83%. The clinical implications of these findings suggest that 54% of patients could have avoided diagnostic ICA based on PCD‐CCTA results which would have resulted in a reclassification of the CAD‐RADS score. In another study by Sharma et al., the authors compared the diagnostic accuracy of high‐resolution to UHR and found an improvement from 55% to 80%, highlighting the benefit of UHR imaging [42]. Similar results have also been demonstrated for UHR spectral photon‐counting CT imaging, with Fahrni et al. reporting a sensitivity of 100% (95% CI 79–100), specificity of 90% (95% CI 55–96), and accuracy of 96% for the detection of stenoses >50% [43].

Potential future improvements to stenosis quantification may lie in the simultaneous acquisition of polychromatic and spectral data, the benefit of which has been reported for high‐resolution spectral CTs [44, 45]. A similar solution has recently become available for the only currently available PCD‐CT, albeit at the cost of reduced collimation when UHR and spectral images are acquired at the same time (120 × 0.2 vs. 96 × 0.2 mm) [46]. Other improvements to PCD‐CCTA may soon be available in the form of artificial intelligence. Brendel et al. explored the integration of artificial intelligence as a pre‐reading tool for radiologists for the detection of CAD and found that it improved workflow efficiency [47]. However, the relatively low positive predictive value (64.8% per patient, 53.8% per vessel) caused by a high rate of false positives limits autonomous use and necessitates human oversight.

**FIGURE 5:**
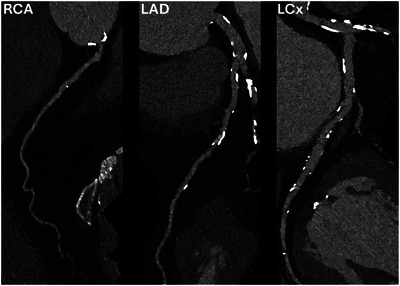
Coronary computed tomography angiography of a 74‐year‐old male performed on a photon‐counting detector (PCD)‐CT using the ultrahigh‐resolution detector mode and reconstructed to a slice thickness of 0.2 mm. Even with extensive coronary calcifications (coronary artery calcium score: 3497 Agatston units), PCD‐CT allows sufficient diagnostic confidence to assess coronary artery lumen patency. LAD, left coronary artery; LCx, left circumflex artery; RCA, right coronary artery.

**FIGURE 6:**
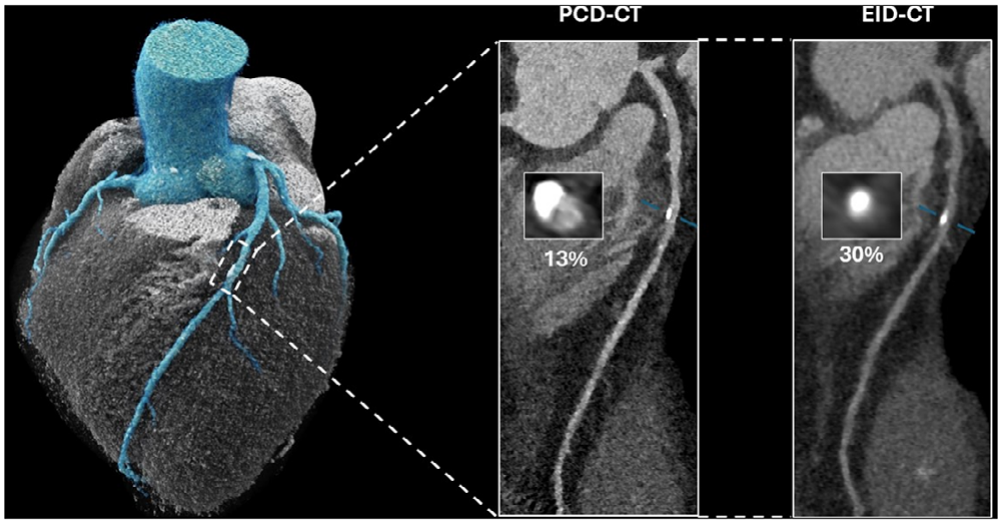
Stenosis of the middle left anterior descending artery in a 69‐year‐old male. Overestimation of the diameter stenosis on the energy‐integrating detector (EID)‐CT compared to the photon‐counting detector (PCD)‐CT using the ultrahigh‐resolution detector mode and reconstructed to a slice thickness of 0.2 mm, is most likely due to extensive blooming artifacts which obscure the true lumen boundary.

In summary, a decrease in blooming artifacts on PCD‐CTs compared to conventional EID‐CTs contributes to more accurate CT stenosis measurements when referenced against the gold standard of ICA. This allows PCD‐CTs to overcome a known drawback of EID‐CTs: the overestimation of stenoses. Figure 5 shows a patient who successfully underwent PCD‐CCTA with a CAC score of 3497 Agatston units, a coronary calcium burden that typically tremendously limits diagnostic EID‐CCTA's capabilities. Another example demonstrated a 17% reduction in diameter stenosis measurements from EID‐CT to PCD‐CT in the same patient (Figure 6).

#### 3.2.2 Plaque Quantification and Characterization

Although ICA remains the gold standard for stenosis assessment, it offers limited information on plaque composition. For detailed insights into plaque composition, invasive and costly methods such as intravascular ultrasound or optical coherence tomography are required. In contrast, CCTA provides data on the lumen as well as vessel wall, allowing for plaque characterization. Currently, coronary lumen stenosis is the main CCTA criterion used to guide patient management, although it has been found that plaque volume, more specifically, non‐calcified plaque volume, is a superior predictor of MACE than lumen stenosis or clinical risk profile [48, 49].

The first‐in‐human study to assess the utility of UHR PCD‐CT found that UHR reconstructions (0.2 mm/sharp Bv64 kernel) yielded a smaller median plaque volume than reconstructions at the reference standard (0.6 mm/smooth Bv40 kernel) at a median of 18.1 mm^3^ versus 23.5 mm^3^, respectively [50]. An explorative study by Mergen et al. (*n* = 20) demonstrated that UHR PCD‐CT CCTA of calcified coronary arteries (CAC score: median 479 [IQR 251–834]) is feasible, yielding excellent image quality, high sharpness, and reduced blooming, recommending reconstruction parameters of a 512 × 512 matrix size with a 200 × 200 mm^2^ field of view, and the use of a sharp Bv64 kernel [51]. An ex vivo study of advanced atherosclerotic carotid plaques confirmed the ability of PCD‐CT to distinguish between different plaque components such as lipids, thrombus, fibrous caps, and necrosis [52].

Overall, the use of UHR PCD‐CT for plaque characterization may lead to improved visualization of different plaque components such as fibrotic or lipid‐rich plaques, potentially improving overall risk stratification. Although, currently, there are no invasive validation methods available for UHR PCD‐CT‐based plaque characterization.

#### 3.2.3 Coronary Stents

The assessment of stent patency is a known issue for CTs due to blooming and beam hardening artifacts. A meta‐analysis by Dai et al. found that CCTA may be unreliable for patients with smaller stents, bifurcating stents, or stents with a thick strut structure [53]. A study on the latest, third‐generation dual‐source CTs collaborated by ICA found that EID‐CTs could rule out in‐stent restenosis in about a third of all symptomatic patients with about a quarter of CCTAs being inconclusive [31].

Initial phantom experiments demonstrated PCD‐CTs superiority to EID‐CT regarding in‐stent lumen delineation using similar reconstruction parameters [54, 55, 56, 57, 58, 59]. Further recent developments suggest that the use of combined UHR and spectral imaging may further improve stent imaging on PCD‐CT [46]. One of the first human PCD‐CT studies demonstrated that in vivo stent imaging benefited from PCD technology due to improved image quality and artifact handling [60].

Consecutively, larger studies such as by Qin et al., encompassing 69 patients, showcased significantly improved diagnostics and stent visualization of UHR PCD‐CT compared to standard resolution with larger in‐stent diameters. Diagnostic accuracy improved from 78.3% (standard resolution) to 88.0% (UHR) with ICA as the reference standard [61]. The authors concluded that sharper kernels (Bv72/Bv76) combined with UHR yielded the most optimal results for stent visualization.

In a study by Hagar et al. with 18 patients and 44 stents, the authors observed a 100% sensitivity, 87.2%–92.3% specificity, 50%–62.5% positive predictive value, and 100% negative predictive value, with an excellent inter‐reader agreement for the assessment of stent patency (90.1%, Cohen's *κ*: 0.72) when comparing UHR PCD‐CT CCTA to ICA [62]. As with the previous publication, sharper kernels (in this case Bv60) combined with UHR and a quantum iterative reconstruction strength of three led to the best results.

**FIGURE 7:**
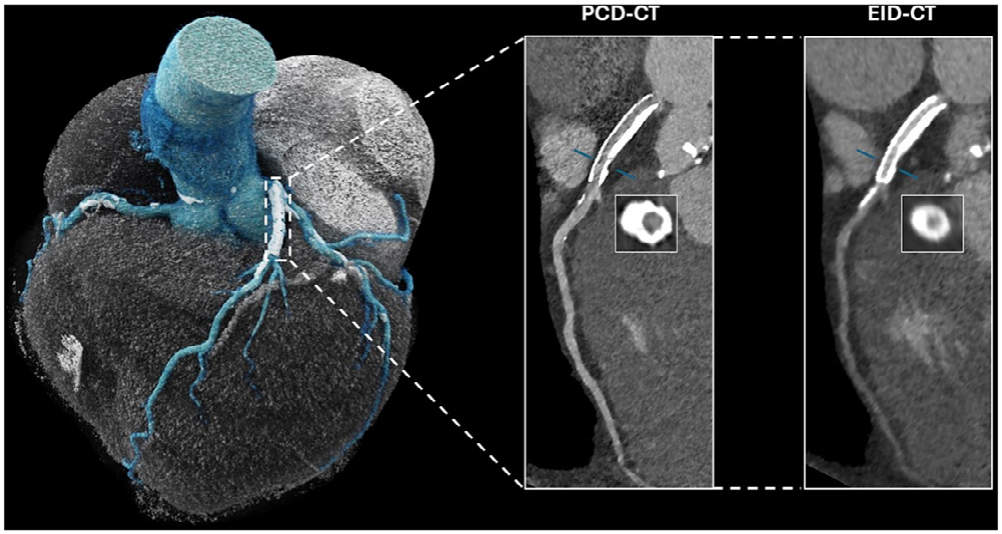
Stent along the left anterior descending artery in a 76‐year‐old male. Reduced blooming artifacts in the photon‐counting detector (PCD)‐CT image (acquired using the ultrahigh‐resolution detector mode and reconstructed to a slice thickness of 0.2 mm) compared to the energy‐integrating detector (EID)‐CT allows better in‐stent lumen visualization.

Larger, randomized studies are still needed to assess the impact of UHR coronary stent PCD‐CT imaging may have on patient workflows. Ideally, UHR PCD‐CT would improve diagnostic confidence to the point that unnecessary diagnostic ICAs could be avoided. A comparison of EID‐ versus PCD‐CT stent imaging is provided in Figure 7.

### 3.3 Tissue Characterization

Tissue characterization has been traditionally viewed as an MRI‐dominated field. Routinely acquired spectral information with improved spatial resolution has now opened up the field to PCD‐CT. As most tissue characterization‐related topics will rely on the use of spectral data and the reconstruction of VMIs, this review will briefly touch upon the topics concerning UHR.

#### 3.3.1 Epicardial Adipose Tissue and Pericoronary Adipose Tissue

Both epicardial adipose tissue (EAT) and pericoronary adipose tissue (PCAT) fat attenuation index (FAI) are thought to be metabolically active tissues and have been associated with cardiovascular disease and CAD [63, 64, 65]. CT allows the non‐invasive evaluation of EAT attenuation and volume as well as PCAT FAI, but both are influenced by acquisition and reconstruction parameters [66].

One of the first PCD‐CT studies on a phantom model and in vivo scans was performed by Mergen et al. using high resolution and VMIs [67]. Their findings collaborated that EAT measurements are affected by VMI levels as well as contrast enhancement. The same research group later went on to validate similar findings for PCAT FAI in an animal model, observing that findings were influenced by VMI level, lumen attenuation, and reconstruction kernel when comparing standard resolution EID‐ and PCD‐CT [68]. An intra‐individual study comparing EID‐ versus PCD‐CT EAT measurements demonstrated that both EAT attenuation and EAT volumes were significantly different between UHR PCD‐CT (slice thickness 0.2 mm) compared to standard resolution PCD‐CT (0.6 mm), and EID‐CT (0.6 mm) [69]. Interestingly, the tendencies observed in all three groups were similar on an intra‐individual level, highlighting the utility of EAT measurements with UHR PCD‐CT but also the need for the development of standardized protocols. Although not performed at UHR, a study by Lisi et al. also found that PCAT FAI is highly dependent on the used reconstruction kernel and level of iterative reconstruction, further highlighting the need for a standardization for the implementation of PCD‐CT examinations for EAT and PCAT assessment [70].

### 3.4 Other Applications of UHR PCD‐CT

The use of UHR PCD‐CT is not limited to stenosis measurements or stent assessments. Undoubtedly, will all topics of cardiac imaging benefit from the improvements that are inherent to UHR PCD‐CTs. A few examples are discussed in the following paragraphs.

#### 3.4.1 Radiomics

Radiomics is a rapidly evolving field of research that seeks to extract quantitative metrics from medical images. Unsurprising, the application of radiomics has also reached cardiac CT research in recent years and with that PCD‐CT. A pilot study on the radiomics features of fruit found fundamental differences when controlling for acquisition and reconstruction parameters between EID‐ and PCD‐CT at standard‐resolution [71]. Ayx et al. were one of the first to use standard resolution cardiac PCD‐CT for the assessment of myocardial radiomics features compared to EID‐CT [72, 73]. Although they concluded that some first‐order radiomics features of the left ventricle were comparable between the two technologies, it was discovered that higher‐order features were different potentially due to the improved spatial resolution, discrimination of photon energies, and better signal‐to‐noise ratios (SNR). The same team later used standard resolution PCD‐CT to demonstrate satisfactory feature stability in an organic phantom model [74], before moving on to in vivo experiments assessing periaortic radiomics features, finding a correlation between changes in periaortic adipose texture and the CAC score on 2 mm thick images [75]. Wolf et al. demonstrated the effects of VMI on radiomics, finding that VMIs below 90 keV decreased feature stability on high‐resolution [76], while Tremamunno et al. went on to explore intra‐individual myocardial radiomics features between standard resolution EID‐ and UHR PCD‐CT [77]. Their findings highlighted that most features remained stable between the two detector technologies at certain VMIs with improved inter‐reader reproducibility on the PCD‐CT scanner.

#### 3.4.2 CT‐Fractional Flow Reserve

Although percutaneous coronary intervention has long been the primary treatment for obstructive CAD, studies indicate it may be ineffective or even harmful in patients without hemodynamically significant coronary stenoses [78]. Lesion‐specific, non‐invasive CT fractional flow reserve (CT‐FFR) assessment aims to provide vital information of stenosis hemodynamics which until only recently, was only possible via invasive ICA. As these algorithms were developed for standard resolution EID‐CTs, the reproducibility of CT‐FFR results on PCD‐CTs remained an open question.

The first intra‐individual comparison of standard resolution CT‐FFR between EID‐ and PCD‐CT demonstrated strong agreement between the two technologies, proving feasibility [79]. A further study by Vecsey‐Nagy et al. comparing 34 patients examined on both EID‐CT and PCD‐CT scanners showed excellent inter‐scanner agreement for CT‐FFR values (ICC: 0.93 [0.90–0.95]) [80]. More importantly, UHR PCD‐CT FFR values were significantly higher than EID‐CT on both a vessel (0.58 ± 0.23 vs. 0.55 ± 0.23, *p* < 0.001, respectively) and patient level (0.73 ± 0.23 vs. 0.70 ± 0.22, *p* < 0.001). This led to a reclassification of two patients to hemodynamically non‐significant stenosis on PCD‐CT from hemodynamically significant on EID‐CT. These findings highlight the growing importance of CT‐FFR and its potential to replace invasive FFR measurements as UHR PCD CT‐FFR becomes more reliable and reproducible, and is validated with prospective studies.

#### 3.4.3 Valve Imaging

The CT evaluation of patients before TAVR is an important pre‐interventional step to ensure patient suitability before the costly procedure. A recent large, multicenter study of asymptomatic patients with severe aortic stenosis evaluated clinical surveillance compared to early TAVR [81]. Results showed a superior clinical outcome of early TAVR with reduced rates of unexpected hospitalizations. If these findings find their way into clinical guidelines, the number of pre‐ and post‐TAVR scans is guaranteed to increase and with them the number of people undergoing the procedure.

Boccalini et al., in a phantom study, found that objective and subjective image quality were improved for PCD‐CTs compared to dual‐energy, dual source EID‐CTs by reducing artifacts and increasing spatial resolution [82]. A case series by van der Bie et al. showcased the improved PCD‐CT image quality in vivo, even demonstrating the ability to assess hypoattenuating leaflet thickening [83]. Dirrichs et al. conducted a study with 300 patients for TAVR planning using an EID‐ and PCD‐CT [84]. Although PCD‐CT CNR (47.3 ± 14.8 vs. 59.3 ± 21.9, *p* < 0.001) and SNR (33.0 ± 10.5 vs. 47.3 ± 16.4, *p* < 0.001,) were lower than for the EID system, subjective image quality was much higher (4.8 vs. 3.3, *p* < 0.001) and rated as excellent in 160/202 (79.2%) of PCD‐CT versus 5/100 (5%) of EID‐CT cases. More importantly, the rate of non‐diagnostic scans was reduced from 11/100 (11%) for the EID‐CT to only 1/202 (0.5%) on the PCD‐CT.

Hagar et al. further highlighted the importance of spatial resolution and acquisition technique when they compared retrospective ECG‐gated UHR PCD‐CCTA (collimation: 120 × 0.2 mm) with high‐pitch spiral ECG‐triggered CCTA with spectral capabilities (collimation: 144 × 0.4 mm) [85]. Retrospective PCD‐CCTA resulted in a higher effective dose than the high‐pitch spiral acquisitions (12.6 mSv [10.3–15.0] vs. 4.1 mSv [3.7–5.1]), *p* < 0.001), but showcased significantly better image quality (median score, 4 [IQR, 3–4] vs. 3 [IQR, 2–3]; *p* < 0.001).

## 4 Radiation Dose

The total number of CT examinations worldwide shows an increasing trend, a study from 2020 estimates that approximately 300 million CT scans are performed per year worldwide and due to increase by 4% per year [86]. The resulting radiation exposure from this is not non‐significant, according to the World Health Organization, approximately 20% of the annual absorbed radiation by humans is caused by medical imaging [87]. In recent years several dose‐reducing applications have been developed including dual‐energy CT, automatic exposure control, ECG‐triggered acquisitions, and low‐dose examination protocols [88, 89].

PCD‐CTs allow for a lower total CT dose index compared to EIDs with a reduction of radiation dose ranging from approx. 30% to 66% by filtering out electronic noise and efficient x‐ray photon weighting [90, 91, 92]. Dirrichs et al. highlighted the advantages of standard resolution detector mode PCD‐CT acquisitions in a study involving 113 neonates where at similar mean effective doses (PCD‐CT: 0.50 mSv, EID‐CT: 0.52 mSv; *p* = 0.47) subjective image quality (4.17 vs. 3.16; *p* < 0.001), SNR (46.3 ± 16.3 vs. 29.9 ± 5.3; *p* *= *0.007), and CNR (62.0 ± 50.3 vs. 37.2 ± 20.8; *p* = 0.001) were significantly improved [93]. A study by Martinez et al. further highlighted the benefit of acquiring images using the UHR detector mode (collimation of 120 × 0.2 mm) compared to the standard resolution detector mode (144 × 0.4 mm) [94]. They observed a potential dose reduction of up to almost 90% between the two acquisition modes, emphasizing that acquisitions should always be performed using the UHR detector mode to take advantage of the intrinsic noise and dose reduction.

## 5 Outlook and Clinical Implications

Although PCD‐CT is not a new imaging modality and still harbors some drawbacks of conventional CT such as radiation exposure, it did innovate the technology to a new degree. UHR PCD‐CT imaging has facilitated visualization of structures in the micrometer domain, high‐quality intra‐stent imaging enabling confident diagnosis, and the reduction of common artifacts allowing us to see more.

Recently, a large retrospective study encompassing 7833 subjects found that patients who underwent CCTA on a PCD‐CT were less likely to be referred to ICA, and the ones that did receive ICA were more likely to undergo revascularization procedures than patients scanned on EID‐CT systems [95]. Vecsey‐Nagy et al. further explored the consequences of decreased follow‐up testing through a cost‐effectiveness analysis which found that PCD‐CT has the potential to reduce costs through a decrease in follow‐up tests and associated complications, leading to potential savings of $794.50 ± 18.50 per patient [96].

Although PCD‐CCTA has not been explicitly incorporated into clinical guidelines, it is just a question of time before publications such as the previously mentioned will enable PCD‐CT to outright replace EID‐CTs. Until then, further prospective, multicenter studies are required to facilitate the jump of PCD‐CCTA to become the new reference standard. The incorporation of always‐on spectral imaging with UHR might even allow PCD‐CT to integrate angiographies with plaque characterization and allow for better risk stratification.

## 6 Conclusion

Although we are still at the beginning of the PCD‐CT era, the advantages of this technology over previous generation CT scanners are already evident. UHR imaging with photon energy discrimination leads to superior, more confident diagnostic capabilities resulting in fewer non‐diagnostic examinations. The downgraded reclassification of disease severity through more accurate imaging combined with plaque characterization has the potential to improve risk stratification and reduce unnecessary diagnostic ICAs and improve preselection for interventional procedures. It remains to be seen how PCD‐CCTA will be integrated into guidelines and how it will shape patient outcomes.

## Data Availability

Data sharing is not applicable to this article as no new data were created or analyzed in this study.
